# Multifunctional Coatings for Robotic Implanted Device

**DOI:** 10.3390/ijms20205126

**Published:** 2019-10-16

**Authors:** Caterina Cristallini, Serena Danti, Bahareh Azimi, Veronika Tempesti, Claudio Ricci, Letizia Ventrelli, Patrizia Cinelli, Niccoletta Barbani, Andrea Lazzeri

**Affiliations:** 1 Institute for Chemical and Physical Processes, IPCF ss Pisa, CNR, c/o Largo Lucio Lazzarino, 56126 Pisa, Italy; niccoletta.barbani@unipi.it (N.B.); andrea.lazzeri@unipi.it (A.L.); 2 Department of Civil and Industrial Engineering, DICI, University of Pisa, Largo Lucio Lazzarino, 56126 Pisa, Italy; serena.danti@unipi.it (S.D.); b.azimi@ing.unipi.it (B.A.); patrizia.cinelli@unipi.it (P.C.); 3 INSTM, National Interuniversity Consortium of Materials Science and Technology, Via G. Giusti 9, 50121 Firenze, Italy; v.tempesti@outlook.it (V.T.); clauk8@gmail.com (C.R.); letiventi@gmail.com (L.V.)

**Keywords:** composite, poly(3-hydroxybutyrate-co-3-hydroxyvalerate) fibers, physico-chemical characterization, hydrophilic hydrogels, synthetic primers, biological assay, functional tests, drug delivery

## Abstract

The objective of this study was the preparation and physico-chemical, mechanical, biological, and functional characterization of a multifunctional coating for an innovative, fully implantable device. The multifunctional coating was designed to have three fundamental properties: adhesion to device, close mechanical resemblance to human soft tissues, and control of the inflammatory response and tissue repair process. This aim was fulfilled by preparing a multilayered coating based on three components: a hydrophilic primer to allow device adhesion, a poly(vinyl alcohol) hydrogel layer to provide good mechanical compliance with the human tissue, and a layer of poly(3-hydroxybutyrate-co-3-hydroxyvalerate) fibers. The use of biopolymer fibers offered the potential for a long-term interface able to modulate the release of an anti-inflammatory drug (dexamethasone), thus contrasting acute and chronic inflammation response following device implantation. Two copolymers, poly(vinyl acetate-acrylic acid) and poly(vinyl alcohol-acrylic acid), were synthetized and characterized using thermal analysis (DSC, TGA), Fourier transform infrared spectroscopy (FT-IR chemical imaging), in vitro cell viability, and an adhesion test. The resulting hydrogels were biocompatible, biostable, mechanically compatible with soft tissues, and able to incorporate and release the drug. Finally, the multifunctional coating showed a good adhesion to titanium substrate, no in vitro cytotoxicity, and a prolonged and controlled drug release.

## 1. Introduction

Notable progress in recent years has been made in biopolymers, synthetic polymers, and advanced composite materials. The exploitation of chemical, biological, and engineering methods has led to the generation of new polymeric productions by combining the polymer synthesis and polymer chemical modification for advanced applications in relevant industrial environments.

The use of bio-based polymers, such as poly(hydroxyalkanoate)s, has increased dramatically in various production fields including packaging, automotive, civil engineering, cosmetic, and medicine [1]. An interesting new development considers the modification of polymer surfaces and interfaces, generating, for example, hybrid coatings using organic or inorganic nanostructures to improve the surface properties of traditional polymeric materials [2,3]. A central goal is to obtain a deeper understanding of the relationship between the microstructure and macrostructure of composites, leading to the development of modified and new polymers with unique and specific material properties.

Research in biopolymers and synthetic polymers has developed in the biomedical field, contributing to the production of new biocompatible materials. In this general context, there is a large interest in the development of implantable robotic devices to allow patients with severe hormonal alterations or chronic pain to benefit from an automatic and effective administration of specific drugs [4]. However, long-term electronic implants present specific challenges, including stable performance and materials having poor bio- and cytocompatibility, resulting in immune reactions and infections [5]. For implantable materials, it is important to address the interface between the device and human body. To allow a better interaction of the medical device with the biological system surrounding it, often, polymeric coatings are applied [6]. 

A successful coating must be biocompatible, nontoxic and sterilizable, have sufficient mechanical properties, be durable under conditions of use, and adhere to the device. Hydrophilic coatings, prepared using polymers with functional groups able to absorb water, such as amino, hydroxyl, or carboxyl groups, are widely used to produce coatings on metallic or plastic substrates [7]. Interestingly, coatings based on hydrogels can provide additional advantages such as good biocompatibility, good wetting, and low friction, making them excellent candidates as coating for soft tissue implants [8].

The objective of this study was to obtain a multifunctional coating for an innovative, fully implantable device. The coating was designed to have three fundamental properties: adhesion to device, close mechanical resemblance to human soft tissues, and control of the inflammatory response and tissue repair process. 

To achieve an effective adhesion to the device, two acrylic primers in the form of thin films were studied and characterized. Poly(vinyl acetate-acrylic acid) P(VAc-*co*-AA) copolymer was synthetized by radical polymerization, starting from vinyl acetate (VAc) and acrylic acid (AA); a successive hydrolysis allowed the synthesis of poly(vinyl alcohol-acrylic acid) P(VA-*co*-AA) copolymer [9,10]. A thin layer of P(VA-*co*-AA) was dehydro-thermally cross-linked [11]. The primers act as intermediates to improve the adhesion force between the device substrate, metallic or polymeric, and the real coating; a second layer of poly(vinyl alcohol) (PVA) hydrogel was prepared by repetitive freezing and thawing of aqueous PVA solutions [12] and loaded with dexamethasone (Dexa). A third layer of biodegradable drug-loaded ultrafine fibers was obtained by electrospinning of PHBHV solutions containing Dexa. The copolymer formation was characterized and evaluated by FT-IR, differential scanning calorimetry (DSC), and thermal gravimetric analysis (TGA) analysis. The components of the coating were characterized from morphological (scanning electron microscopy, SEM), mechanical (dynamic mechanical analysis, DMA), chemical (FT-IR chemical imaging), biological (cytotoxicity test), and functional (high permeation liquid chromatography, HPLC, adhesion tests) properties.

## 2. Results and Discussion

### 2.1. Synthesis and Characterization of Primers

In order to improve the adhesion of coating to the device’s external substrate, two copolymer acrylic primers were identified and synthetized. A first copolymer of vinyl acetate (VAc) and acrylic acid (AA) was successively treated to obtain the second copolymer of vinyl alcohol (VA) and AA. Chemical structure of the copolymers was confirmed by FT-IR spectroscopy, the correlation of spectra with corresponding homopolymers allowed us to obtain a chemical composition of VAc (or VA) and AA of 70:30 wt.%, according a method reported in our previous work [9]. 

#### 2.1.1. Differential Scanning Calorimetry (DSC)

**Figure 1:**
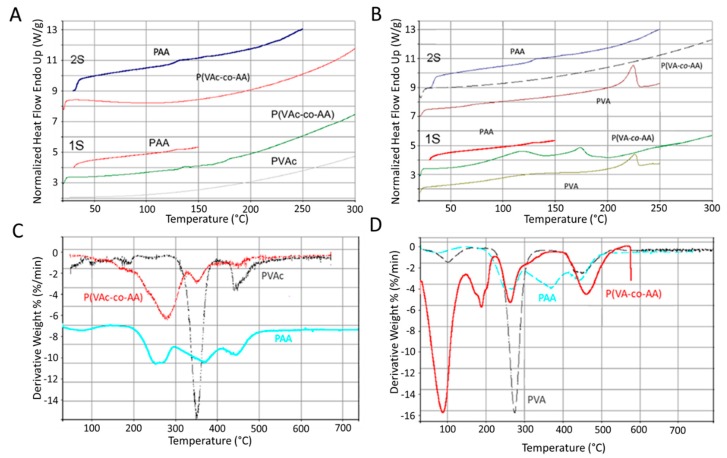
Differential scanning calorimetry (DSC) thermograms of P(VAc-*co*-AA) and pure components, first and second scan (**A**); DSC thermograms of P(VA-*co*-AA) and pure components, first and second scan (**B**); TGA/DGA traces of P(VAc-*co*-AA) and pure components (**C**); TGA/DGA traces of P(VA-*co*-AA) and pure components (**D**).

The thermogram of P(VAc-*co*-AA) shows, in the first scan, the event corresponding to the glass transition (Tg) of PAA segment, while any signal was detected for the PVAc component, as confirmed by the thermogram of pure PVAc (Figure 1A). Thermogram of P(VA-*co*-AA), in the first scan, shows an enlarged event near to 100 °C, due to its elevated hydrophilicity, and an endothermic event near to 180 °C, corresponding to the crystalline fraction of the PVA segment, with a result lower than the same event registered for pure PVA (Figure 1B). In the second scan, both copolymers do not reveal any significant event. This fact can be associated to a crosslinking effect of copolymer chains; on the contrary, the Tg for pure PAA and melting temperature (Tm) for pure PVA were detected around 220 °C. The crosslinking effect of the obtained copolymer is an important indication that the dehydro-thermal treatment, used then to make the copolymer primer films stable in an aqueous environment, can be suitable and effective.

#### 2.1.2. Thermogravimetric Analysis (TGA)

The derivative TGA (DGA) of pure PVAc showed a first peak of degradation at 350 °C and a second event at 470 °C. TGA/DGA of P(VAc-*co*-AA) points out a degradation peak at 350 °C, lower than for pure PVAc, probably due to the high content of PVA. A second peak at 470 °C due to PVAc segment and an enlarged event in the range of 200–300 °C due to PAA segment thermal degradation were observed (Figure 1C). TGA of pure PVA showed a weight loss at 100 °C due to water content, a more intense weight loss at 280 °C, and a residual peak at 475 °C. TGA of P(VA-*co*-AA) showed a large water loss at 100 °C, in agreement with DSC results; a second degradative event between 200–300 °C, a range of temperatures where both PVA and PAA degrade; and a last loss around 480 °C. In the copolymer trace, a peak between 100 °C and 200 °C appeared, while this peak was not present in both pure components (Figure 1D). This could be attributable to bound water leading, successively, to the physical crosslinking of the material. The degradation mass loss of the PAA component shifted towards a higher temperature (around 500 °C) due to the presence of hydrogen bond interactions among functional groups of the copolymer matrix. 

#### 2.1.3. FT-IR Chemical Imaging

**Figure 2:**
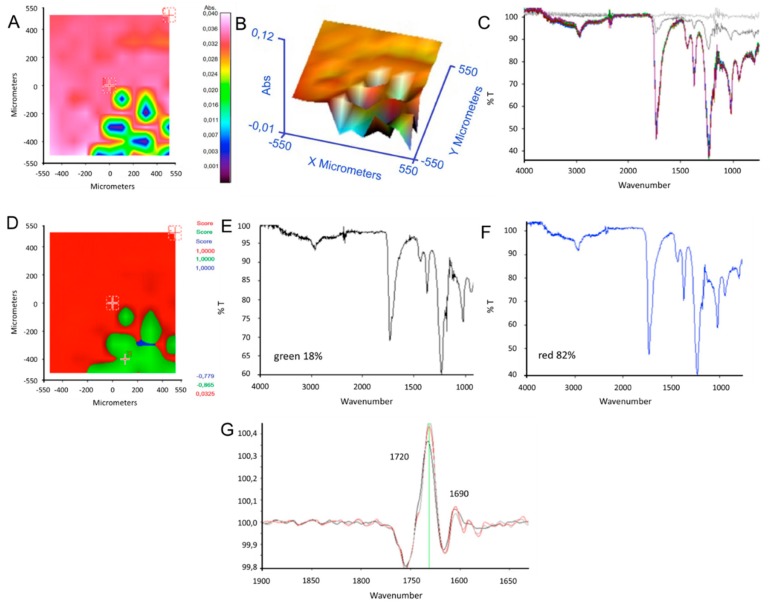
Chemical map in 2D (**A**), in 3D (**B**), and corresponding (**C**) spectra of P(VAc-*co*-AA) primer after deposition on Ti substrate; PCA analysis map (**D**) and spectra corresponding to green area (**E**) and red area (**F**); derivative spectra acquired in green area (**G**).

To evaluate the homogeneity of the copolymer coating on titanium substrates, FT-IR analysis was carried out on the surface of P(VAc-*co*-AA) film after its deposition on a titanium substrate. In Figure 2A–C, a chemical map in 2D and in 3D and corresponding spectra are reported. The presence of typical absorption bands for PVAc at 1720 cm^−1^ and PAA groups at 1690 cm^−1^ is evident over the whole area examined. Moreover, the disappearance of monomers was confirmed by the intensity change of absorption bands corresponding to vinylic groups at 1600 cm^−1^ and 1400 cm^−1^. In Figure 2D the PCA analysis points out two distinct homogeneous spectral groups; spectra (Figure 2E,F) corresponding to two different sections of the surface show in both areas the presence of peaks associated with copolymer composition. The visibly different area in the lower right corner than the rest of the surface indicates a lower covering of the material, which is however present, as it can be seen from the derivative spectra acquired in this area (Figure 2G). This shows both the peak at 1690 cm^−1^ due to PVAc groups and the peak at 1720 cm^−1^ due to carboxylic groups stretching the PAA segment. 

**Figure 3:**
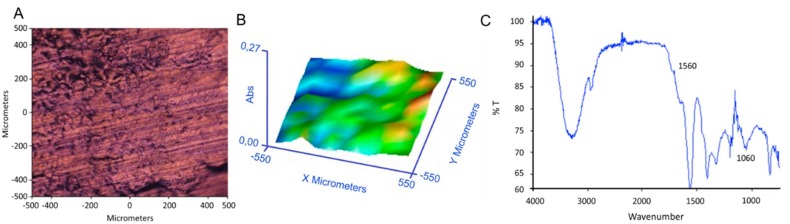
(**A**) Optical image of coating of P(VA-co-AA) onto Ti substrate; (**B**) 3D chemical map; (**C**) reference spectrum of copolymer after deposition.

In Figure 3, optical image, 3D chemical map, and reference spectrum of P(VA-*co*-AA) primer film after deposition on Ti substrate are reported. The results confirm a complete and homogeneous coverage of copolymer on substrate. The spectrum of the copolymer showed the presence of typical absorption bands for PVA and PAA: OH stretching absorption at 3300–3400 cm^−1^, CH stretching at 2940 cm^−1^, carboxylic groups stretching at 1720 cm^−1^, carboxylate groups stretching at 1560 cm^−1^, and CO stretching at 1262 cm^−1^ and 1060 cm^−1^. 

#### 2.1.4. Biological Assays

The cell viability tests performed on the P(VAc-*co*-AA) copolymer showed well spread and elongated fibroblasts at 24 h, similar to the control. Resazurin reduction at 24 h was 99.79% ± 4.10% for the control and 99.30% ± 4.20% for the copolymer.

**Figure 4:**
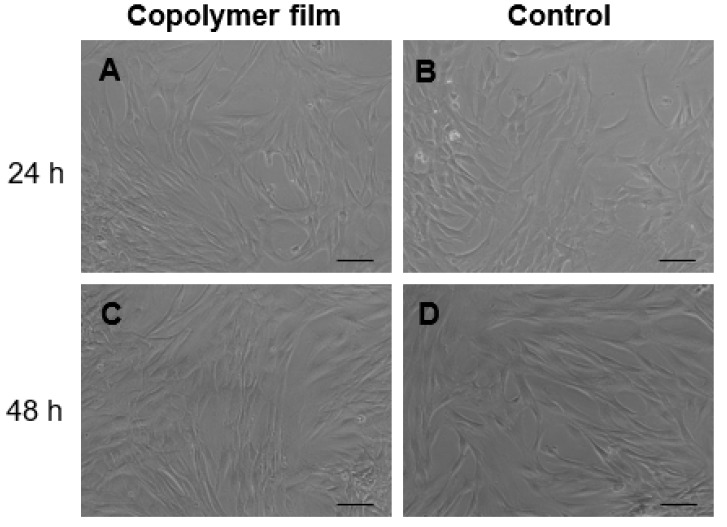
Results of the indirect cytotoxicity assay performed on P(VA-co-AA) copolymer film: micrographs acquired using a light inverted microscope of human dermal fibroblasts (hDFs) (**A**,**C**) treated with and (**B**,**D**) without conditioned culture medium (CM) for (A,B) 24 h and (C,D) 48 h. Scale bar is 100 µm.

The metabolic activity of cells on P(VA-*co*-AA) copolymer film was evaluated using Alamar blue assay; at 48 h the percentage of viability was 74.17% ± 4.11% for the control and 69.69% ± 7.21% for copolymer. In Figure 4, the morphology of cells seeded on P(VA-co-AA) copolymer film appeared similar to 2D cells, both at 24 and 48 h, indicating a good compatibility of synthetized copolymer with primary human dermal fibroblasts (hDFs). 

### 2.2. Production and Characterization of PVA Hydrogel

In regards to the external coating layer, PVA hydrogels represent materials capable of satisfying the requirements of biocompatibility, mechanical compatibility with soft tissues (considering that the device will be inserted into the abdominal cavity), and an optimal ability of incorporating and releasing drugs (i.e., dexamethasone). The hydrogels were prepared with the freeze–thaw method, which is intrinsically nontoxic as it does not require the use of chemical crosslinkers. It deals with a physical treatment, which does not require high-energy radiation, instead it exploits the numerous and synergistic hydrogen bonds occurring between the OH groups of PVA chains and the water molecules forming cross-linking sites, making the PVA macromolecules stable in the aqueous environment [13]. 

#### Mechanical Analysis 

In Table 1, elastic modulus (E’) and loss modulus (E’’) for PVA hydrogel (8% wt.) are reported. Mechanical analysis showed a storage modulus, measured in wet conditions, in the range of values of soft tissues (fat, muscle, skin, cartilage) (0.01–2 MPa). The values obtained are comparable with those reported in literature for adipose and muscle tissues, with which the material will have to interface [14]. 

### 2.3. Production and Characterization of PHBHV Fibers

The third layer is formed of interconnected fibers of PHBHV, a biocompatible and slowly degradable polymer, obtained by electrospinning method. After optimizing the solution parameters and spinning conditions (polymer concentration, solvents, voltage, distance from the collector, and flow rate) the fibers of suitable shape and size were obtained, both before and after loading with Dexa. In the literature, electrospun biodegradable nanofibers have demonstrated capability as carriers for the sustained release of Dexa [15]. 

#### 2.3.1. FT-IR Chemical Imaging

**Figure 5:**
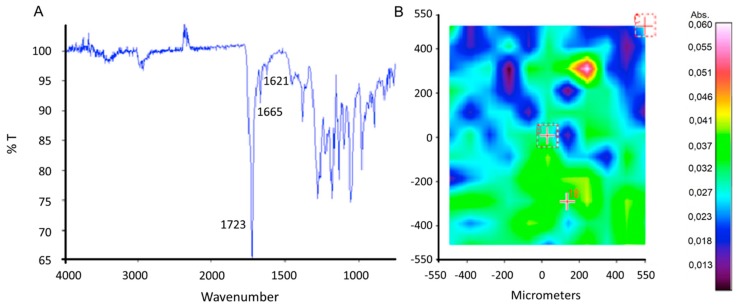
(**A**) Spectrum of PHBHV fiber network loaded with dexamethasone (Dexa); (**B**) correlation map of PHBHV fiber mesh loaded with Dexa in relation to the characteristic spectrum of Dexa.

The presence of Dexa into the PBHBV fibers was confirmed by infrared analysis. In Figure 5A, the medium spectrum of PHBHV fibers loaded with Dexa is reported; the most intense frequency at 1723 cm^−1^ corresponds to carbonyl ester of PHBHV, while the peaks at 1665 cm^−1^ and 1621 cm^−1^ were due to the C=O and C=C (in ring) double bond of Dexa. In Figure 5B, the correlation map, with respect to characteristic spectrum of Dexa, confirms the homogenous distribution of the drug at the level of interconnected fiber mesh.

#### 2.3.2. Release Test from PVA Hydrogel and PHBHV Fiber Composite

**Figure 6:**
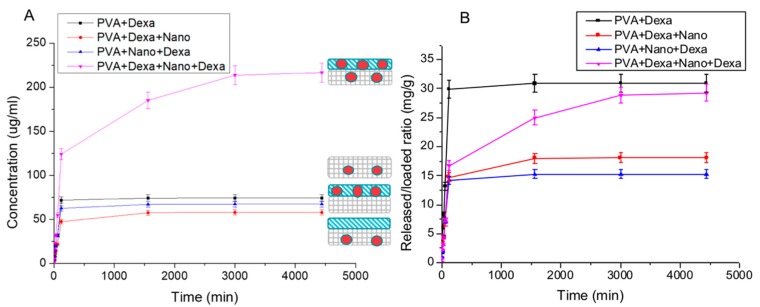
(**A**) Concentration of Dexa released from different samples versus time; (**B**) amount of Dexa released with respect to the initial content of the loaded drug for all samples examined.

In Figure 6, the results of Dexa release from systems formed by assembling PVA hydrogel and fiber mesh are reported. The comparison of release trend was performed among hydrogel PVA loaded with Dexa (PVA + Dexa), hydrogel PVA loaded with Dexa and unloaded fibers (PVA + Dexa + Nano), unloaded hydrogel PVA and fibers loaded with Dexa (PVA + Nano + Dexa), hydrogel PVA loaded with Dexa and fibers loaded with Dexa (PVA + Dexa + Nano + Dexa). As expected, the systems containing both layers (formed by PVA hydrogel and PHBHV fiber mesh) loaded with Dexa showed a more elevated and gradual release of Dexa with respect to systems loaded in a single layer (Figure 6A). Moreover, the values of drug released with respect to initial content of Dexa are reported in Figure 6B. 

The release of Dexa from PVA hydrogel showed a typical burst effect, with a rapid and complete release in the first minutes. The highly porous structure of hydrogel is responsible for this behavior. On the contrary, the release trends for combined systems seem to indicate that the presence of fiber mesh on the upper surface of hydrogel hinders the immediate release of Dexa from the PVA hydrogel. The release of Dexa from fibers on unloaded PVA hydrogel, within 3 days, was reduced due to the presence of molecular interactions with PHBHV fibers. The release trend from the completely loaded system showed a controlled and sustained release that did not reach the stationary phase at 3 days. The tendency for a prolonged release is probably associated with the slow degradation process of PHBHV fibers. In the literature, delivery platforms based on hydrogel/microsphere composites using physically cross-linked PVA containing entrapped Dexa-loaded poly(lactic-co-glycolic acid) (PLGA) microspheres showed potential for coatings for implantable devices [16].

### 2.4. Production and Characterization of Three-Layered Multifunctional Coating

Finally, a three-layered multifunctional coating was assembled on a pretreated titanium substrate. The complete system was characterized using morphological and physico-chemical analysis in order to evaluate the adhesion of different component layers at each interface level. Biological assays on composite coatings were performed in order to evaluate their cytocompatibility, both in the presence and in the absence of drug loaded hydrogel. Moreover, adhesion tests were carried out by immersing the complete system, deposited on titanium substrate, in an aqueous environment at 37 °C under constant agitation. The incubation medium was prepared using both PBS and PBS with added collagenase, MMP-9, in order to simulate in vitro inflammation conditions occurring after device implantation. 

#### 2.4.1. Morphological and Physico-Chemical Characterization of Multifunctional Coating

**Figure 7:**
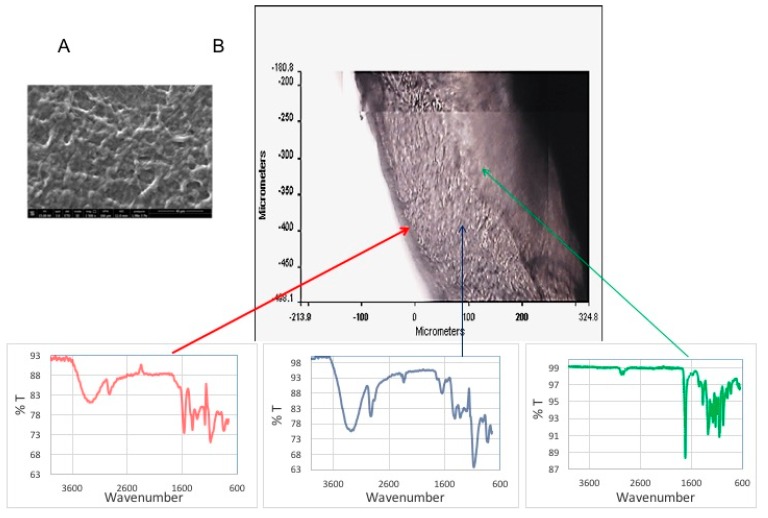
(**A**) SEM image of superior surface of multifunctional coating; (**B**) FT-IR chemical imaging: optical image of a section of three-layered coating (after forced detachment from a titanium substrate), the spectrum to the left of the optical image shows the P(VA-co-AA) bands, the spectrum below shows the PVA bands, and the spectrum to the right of the optical image corresponds to that of PHBHV.

In Figure 7A, a SEM image of the upper surface of a multifunctional coating is reported. The image shows the PHBHV fiber mesh on the PVA hydrogel; it is evident there is a good integration of fibers with the underlying hydrogel coating, thus maintaining the external position of fibers, but at the same time a close contact with the hydrogel. In Figure 7B, an optical image of a section of three-layered coating is reported, showing the three distinct layers, as confirmed by characteristic spectra acquired at each corresponding layer. Moreover, the layers appear closely connected and contiguous to each other.

#### 2.4.2. Release Tests from Final Coating

**Figure 8:**
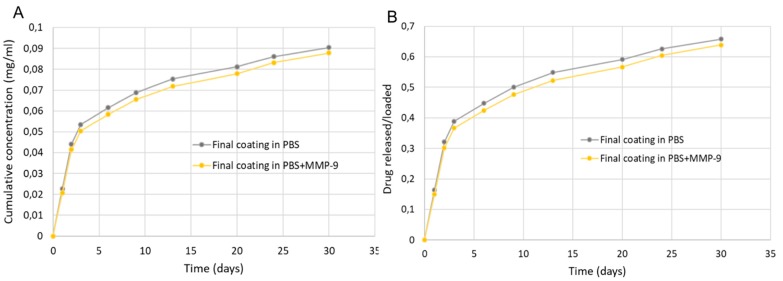
(**A**) Cumulative concentration of Dexa released from composite coating in PBS and in PBS with the addition of MMP-9; (**B**) amount of Dexa released with respect to the initial content of Dexa loaded into both PVA hydrogel and PHBHV fiber mesh.

The release test of Dexa from multilayered coating was carried out both in PBS and in PBS with the addition of MMP-9. The trends of Dexa release are reported in Figure 8.

It is evident that there was a gradual and controlled release of Dexa from the final coating over the whole period of analysis (30 days). The presence of enzymes in the incubation medium did not seem to alter the trend of release of Dexa in the period of analysis. Moreover, it can be seen that the release tended to prolong for longer times, since the trends had not yet reached the equilibrium at 30 days. 

Overall, the release results obtained demonstrate that the choice of loading both the PVA hydrogel and PHBHV mesh with Dexa may accomplish the therapeutic objective of a local, sustained, and prolonged action of the drug, thus maintaining a concrete and extended control on inflammation response after device implant. 

#### 2.4.3. Cytocompatibility Tests of Composite Coating

**Figure 9:**
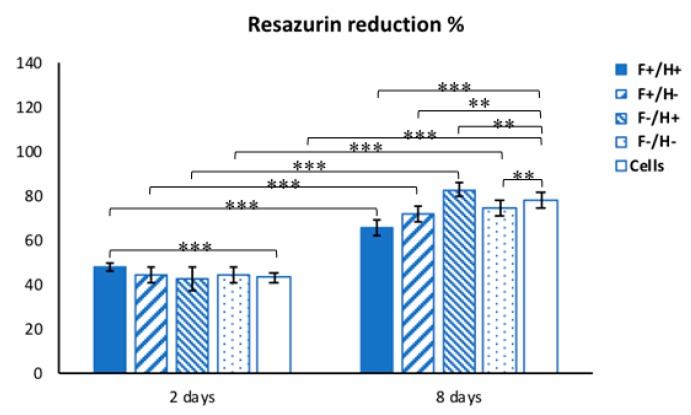
Bar graph showing the metabolic activity (as resazurin reduction percentage, given as average ± standard deviation) of hDFs in contact with the Dexa-loaded and -unloaded fiber/hydrogel composites for 8 days. ** *p* < 0.001, *** *p* < 0.0001.

Cytocompatibility studies were performed by placing the different composites in contact with cell cultures of hDFs for eight days to assess the best composite composition to allow the modulation of fibroblast growth. The results of resazurin assay are reported in Figure 9.

The metabolic activity was evaluated on days two and eight, after hDF cultures were exposed to the four types of fiber/hydrogel (F/H) composites and compared to hDFs grown on tissue culture plastic as positive controls. After two days, the metabolic activity was comparable among the samples, but a statistically significant difference (*p* < 0.0001) was observed between the F+/H+ composite and the control, indicating that the double loading of Dexa enhanced fibroblast viability. Between the two timepoints, an increase of metabolic activity was observed for all the groups (*p* < 0.0001). In particular, on day eight, the lowest metabolic activity was detected in the F+/H+ group, which showed the lowest increase between day two and day eight (17.4% ± 5.3%) and the highest statistically significant difference with the control at the endpoint (*p* < 0.0001).

**Figure 10:**
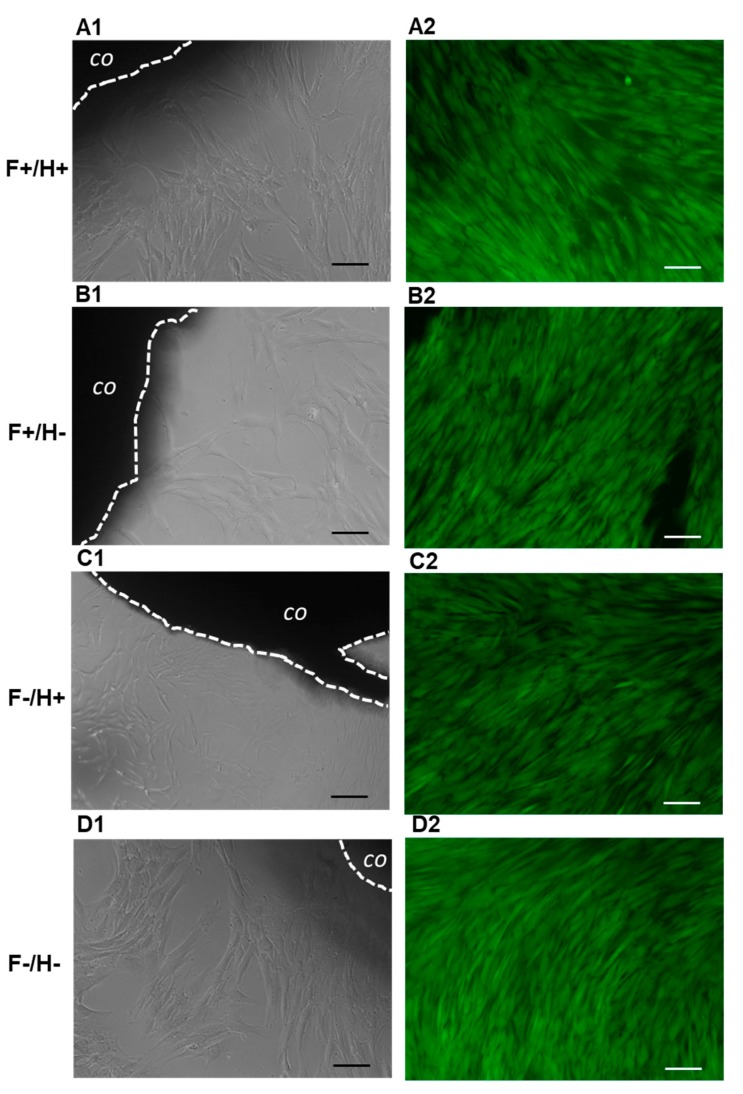
Micrographs showing fiber/hydrogel (F/H) composites in contact with hDFs, as observed via inverted microscopy on day 5 (column 1), and on day 8 after Calcein AM staining (column 2): (**A1**,**A2**) both fibers and hydrogel loaded with Dexa; (**B1**,**B2**) fibers loaded with Dexa and unloaded hydrogel; (**C1**,**C2**) unloaded fibers and hydrogel loaded with Dexa; (**D1**,**D2**) both fibers and hydrogel unloaded. In the micrographs, “co” indicates the composite. Scale bar is 100 µm.

The results of metabolic activity are supported by live micrographs, taken via light inverted microscopy at the different timepoints. Well-elongated fibroblasts are visible in Figure 10, surrounding the composites on days five and eight, without signs of necrosis.

Overall, the composite coatings are highly compatible with primary hDFs. In particular, the Dexa-loaded composite was able to reduce fibroblast growth with respect to the controls. The use of F+/H+ composite could be useful to modulate the fibrous scar on permanently implanted devices.

#### 2.4.4. Adhesion Tests

Two multifunctional coatings were prepared starting from two different primers deposited on Ti substrate, PVA hydrogel loaded with Dexa and PHBHV fiber mesh loaded with Dexa were subjected to an adhesion test. The results of the adhesion test, in saline buffered solution (PBS pH 7.4) and in PBS added with MMP-9 solution performed for 70 days, are reported in Table 2. The photos of the multifunctional coating after removing it from the incubation medium at the end of test show the maintenance of adhesion on the titanium substrate and the reciprocal connection among layers, independently from the composition of incubation medium, kind of primer used, and presence of Dexa. These results seem to confirm the validity in the choice of both single layer components and the assembling method used for the preparation of composite coatings, as well as of two primers that, thanks to the high content of acrylic groups, are able to maintain a stable contact with the metallic substrate. 

## 3. Materials and Methods 

### 3.1. Preparation of Titanium Substrate

NiTi (titanium alloy) was cut into 10 × 10 × 1 mm plates. The plates were washed in water and dish soap and then rinsed in deionized water and dried at 40 °C. They were then oxidized in NaOH 3 M for 1 h to promote the metal–polymer bond.

### 3.2. Preparation of Acrylic Primers 

#### 3.2.1. Synthesis of P(VAc-co-AA)

P(VAc-co-AA) copolymer was synthetized starting from vinyl acetate (VAc), potassium persulphate (KPS), and acrylic acid (AA). Preweighed amounts of PVA (1 g, 80% hydrolyzed, Sigma Aldrich, Milan, Italy) were put into a round-bottomed flask with 150 mL of water. The flask was stirred and heated at 60 °C in an oil bath. As the water began to steam, 45.5 mL of vinyl acetate monomer (VAc) were added. The monomer was treated with resin to eliminate the inhibitor. A 5% solution of K_2_S_2_O_8_ was added. The reaction was performed under nitrogen atmosphere stirring at 200 rpm with a reflux condenser for 15 min. A mixture of AA (21 mL), water (50 mL), and 5% K_2_S_2_O_8_ solution (5 mL) was prepared and 15 mL of that were added to the reaction. The remaining solution was dripped in the flask within 10 min. The reaction was carried out for 3 h under nitrogen atmosphere and then stopped with hydroquinone. The obtained polymer was purified in chloroform.

#### 3.2.2. Preparation of P(VA-co-AA) through Hydrolysis of P(VAc-co-AA)

The polymer (10 g) was put into a round-bottomed flask and a solvent mixture composed by 200 mL of methanol (MeOH) and 150 mL of tetrahydrofurane (THF) was added. The flask was put under stirring and heated with a reflux condenser for 4–5 h. The solution was left under stirring at room temperature overnight. The hydrolysis was made with sodium metoxide (MeONa) in MeOH. MeONa (5 g) was added to 50 mL of MeOH into a beaker (the dissolution process was very exothermic). Then, 15 mL of this solution were added to the polymer solution and a white precipitate was immediately formed. After 30 min, the remaining methoxide solution (35 mL) was put into the flask. The precipitate was filtered and washed with MeOH. The hydrolized polymer was soluble in hot water.

### 3.3. Preparation of PVA Hydrogel

Commercial powder poly(vinyl alcohol) (PVA, Mw 115000, Sigma Aldrich) was dissolved in deionized water in autoclave to obtain a 8% *w*/*v* solution. Drug-loaded hydrogels of PVA were produced by adding dexamethasone (Sigma Aldrich) (0.01% *w*/*v*) and performing repetitive freezing and thawing of the PVA-Dexa solutions (8 cycles at least).

### 3.4. Preparation of PHBHV Electrospun Fiber Meshes

Poly (3-hydroxybutyrate-co-3-hydroxyvalerate) (PHBHV, Sigma Aldrich) was dissolved in chloroform/methanol (9:1 *v*/*v*) mixture at a polymer/solvent concentration of 15% and stirred at 300 rpm for 12 h at room temperature (RT). For production of dexamethasone-loaded PHBHV fibers, 10wt% of Dexa was added to the solution and stirred at 300 rpm for 12 h at room temperature (RT). Each polymer solution was loaded into a 10 mL glass syringe, fitted with a blunt tip stainless steel needle (21G 3/4”), and placed into a syringe pump (NE-300, New Era Pump Systems, Inc. Farmingdale, NY, USA). The ground terminal of high voltage supply (S1600079 Linari High Voltage, Linari Engineering s.r.l, Pisa, Italy) was connected to the metal needle, while the positive terminal was connected to the collector; 30 kV potential was applied. A static collector made of a plastic plate covered with an aluminum foil, or a cylindrical collector (diameter 8 cm, Linari Engineering s.r.l), were placed at a distance of 15 cm from the tip of the needle. The polymer solution was injected from the needle in the presence of the electric field at a constant flow rate of 0.001 mL/min. All the fabrication steps were performed at RT with relative humidity (RH) of about 46% (if not differently specified). The fiber meshes were kept in an oven at 60 °C overnight to remove solvent traces.

### 3.5. Final Assembly of the Multifunctional Composite System

**Figure 11:**
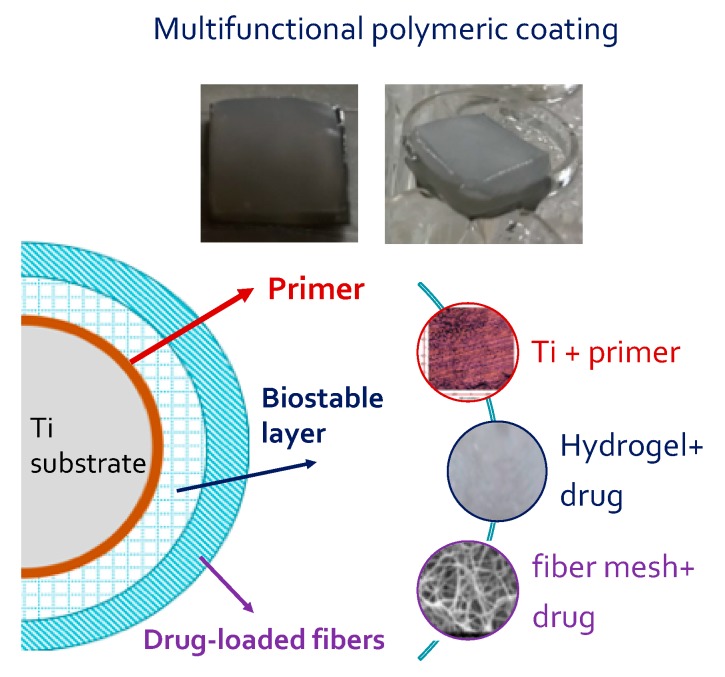
Photos of multifunctional coating onto Ti substrate before and after immersion in incubation medium; schematic representation of final assembly of composite coating.

P(VAc-co-AA) was dissolved in THF to prepare a 1% *w*/*v* solution and P(VA-co-AA) was dissolved in water to prepare a 0.3% w/v solution. Two layers of 250 µL of P(VAc-co-AA) solution or four layers of 250 µL of P(VA-co-AA) solution were stacked up on the oxidized titanium platelets to act as primer between the metal and the PVA hydrogel. The P(VA-co-AA) primer was cross-linked by dehydro-thermal method in a controlled temperature ramp under vacuum conditions: from RT to 50 °C for 2 h, 90 °C overnight, 120 °C for 3 h, and then cooling to 50 °C. The hydrogel layer was produced by deposition of 250 µL Dexa-PVA solution on the selected type of primer. Dexa-PHBHV fiber mesh (1 cm^2^ squares) were put over the hydrogel before the freezing and thawing procedure. The photos of the multifunctional coating before and after immersion in incubation medium and a schematic representation of final assembly are reported in Figure 11. 

### 3.6. Morphological, Thermal, Physico-Chemical, and Mechanical Characterization

Scanning electron microscopy (SEM) was employed to analyze fibers and fibers–hydrogel coating, using a FEI Quanta™ 450 FEG instrument. SEM analysis was done at 15 kV in high-vacuum mode, with manual aperture and 2.5 beam spot size. The samples were sputtered with gold to improve the quality of the analyses. 

The thermal behavior of the P(VAc-co-AA) and P(VA-co-AA) were studied by a differential scanning calorimeter (DSC 7; Perkin Elmer Inc., Waltham, MA, USA), using aluminum pans. Two consecutive scans were carried out on each sample, at scan rate of 10 and 20 °C/min respectively.

Thermogravimetric analysis (TGA-6, Perkin Elmer) was carried out on samples of ca. 10 mg, heated from 25 to 700 °C at a rate of 10 °C/min with a nitrogen purge and ceramic pans.

FTIR analysis in ATR mode (Spectrum 400, Perkin Elmer) was performed on each layer and on the complete system. Infrared spectra were acquired with a Perkin Elmer Spectrum One FT-IR Spectrometer, equipped with ATR objective lens with a penetration depth of less than 1 μm. All spectra were obtained in the middle range (4000–720 cm^−1^) with a resolution of 4 cm^−1^, representing an average of 16 scans. Spectral images were acquired in transmission and in μATR mode using the infrared imaging system Spotlight 300 (Perkin Elmer). The spectral resolution was 4 cm^−1^. The spatial resolution was 100 × 100 μm^2^ in μATR mode and 6.25 μm in transmission. Background scans were obtained from a region of no sample. IR images were acquired with a liquid nitrogen-cooled mercury cadmium telluride line detector composed of 16 pixel elements. Each absorbance spectrum composing the IR images, 16 scans, was recorded for each pixel in the μATR mode using the Spotlight software. Spectra were collected by touching the ATR objective on the sample and collecting the spectrum generated from the surface layer of the sample. The Spotlight software used for the acquisition was also used to preprocess the spectra. IR spectral images were produced by using the absorbance in a given frequency range, 4000–720 cm^−1^. Spectra contained in the spectral images were analyzed using a compare correlation image. The obtained correlation map indicates the areas of an image where the spectra are most similar to a reference spectrum.

DMA analysis was performed with a GABO Eplexor 150 N equipped with compression clamps. The test was conducted at room temperature with a frequency of 1 Hz. Dimension of the specimens used were 17.5 × 15 × 10 mm. PVA hydrogels were tested in two different conditions: static strain of 1%, dynamic strain of 0.5% (initial clamp distance = 10mm) and static strain of 5%, dynamic strain of 1% (initial clamp distance = 10 mm).

### 3.7. Biological Assay

#### 3.7.1. Materials

CaCl_2_ l-glutamine and penicillin/streptomycin, EDTA, trypan blue, and resazurin were purchased from Sigma Aldrich (Milan, Italy). Dulbecco’s modified Eagle’s medium (DMEM), fetal bovine serum (FBS) and phosphate-buffered saline 1×, (PBS), Calcein AM were bought from Gibco by Life Technologies—Thermo Fisher Scientific (Waltham, MA, USA). Diflucan was supplied by Pfizer (New York, NY, USA) and Levoflaxcin (500 mg/100 mL) was bought from Fresenius Kabi (Verona, Italy). Collagenase I was obtained from Worthington Biochemical Corp. (Lakewood, NJ, USA).

#### 3.7.2. Preparation of PHBHV fiber/PVA hydrogel (F/H) composite for cell culture experiments

For cell culture experiments, the composite samples were prepared and handled in sterile conditions. PHBHV fibers, with and without Dexa samples, were cut into 3 cm diameter discs by using a circle stencil template and diamond tip pen, placed at the bottom of sterile 6-well culture plates, and exposed to UV radiation overnight to provide sterilization. The A 10% (*w*/*v*%) PVA solution in double-distilled water was prepared via autoclaving at 121 °C for 1 h (total volume 80 mL) and cooled under a biohood. At the same time, a 0.05% (*w*/*v*%) Dexa solution of D-MEM (Sigma Aldrich, Milan Italy) was prepared and sterile-filtered. D-MEM, either with (Dex+) or without Dexa (Dex-) was added to the PVA solution at 1:4 volume ratio and gently stirred, thus obtaining PVA(Dex+) and PVA(Dex−) solutions, respectively. Each solution was therefore added to each type of fiber samples at 5 mL/well, thus obtaining 4 types of composites, namely PHBHV(Dex-)/PVA(Dex−) (F−/H−), PHBHV(Dex−)/PVA(Dex+) (F−/H+), PHBHV(Dex+)/PVA(Dex−) (F+/H−), and PVA(Dex+)/PHBHV(Dex+) (F+/H+). The 6-well plates were sealed with parafilm and 8 freeze-thawing cycles were performed, consisting of an initial freezing at −20 °C overnight, followed by 7 cycles, each one consisting of thawing at room temperature (RT) for 1 h and freezing at −20 °C for 1 h. Afterwards, the samples were cut into cylinders using a sterile 5 mm diameter puncher.

#### 3.7.3. Ethical Statement

Adult dermal fibroblasts (hDFs) were isolated from waste samples of normal skin derived from contralateral mastectomy to be discarded after surgery. The samples were treated anonymously and in conformity with the Declaration of Helsinki. 

#### 3.7.4. Cell Cultures

Skin specimens were washed with sterile PBS added with antibiotics, cut into 2–3 mm pieces, and washed again. Skin pieces were then treated over night with DMEM, CaCl_2_ 5 mM, 0.25% Collagenase I. After digestion, collagenase was neutralized by adding EDTA and washing twice with DMEM mixed with 10% FBS. Cell suspension was cultured in complete medium and hDFs were used for all the experiments at 4–7 passages. Characterization of hDFs was performed by FACS analysis, checking the expression of fibroblastic markers, such as cadherin-11 and CD-90. Cell culture was carried out in a growth culture medium (CM), consisting of DMEM supplemented with 10% (*v*%) FBS, 2 mM L-glutamine, 100 IU/mL penicillin, and 100 mg/mL streptomycin. Once the 80% confluence was reached, hDFs were trypsinized and viable cells were counted with a hemocytometer, using trypan blue exclusion dye. All the cell culture experiments were performed in a humidified incubator at 37 °C and in 95%/5% air/CO_2_ environment.

#### 3.7.5. Direct Cytotoxicity Tests

HDFs were seeded in 24-well plates at a density of 20,000 cells/well and 1 mL CM. The following day, F/H composites (5 mm diameter) of the 4 different types (*n* = 4) were placed in the wells containing hDFs, one sample per well, and cell culture was continued for 8 days by replacing the CM every 2–3 days. HDFs that were not exposed to the biomaterials were kept in the same culture conditions and used as controls. The samples were monitored daily until the endpoint by reverse microscopy observation and micrographs, which were acquired every 2–3 days to image cell morphology at the periphery of the F/H composites. 

Along the culture time, resazurin dye assay, a nondisruptive metabolic activity test, was performed to check the viability of hDFs. Resazurin is a blue dye, which is irreversibly reduced to the pink colored fluorescent resorufin and used as a REDOX indicator to measure cell metabolic activity. A stock 5 mg/mL solution of resazurin in PBS was freshly prepared and sterile-filtered. At 2 time points, namely day 2 and day 8 after the hDFs were exposed to the composites, the CM was replaced with a 20 µL/mL resazurin working solution prepared in CM. Briefly, samples (*n* = 3) and negative controls (*n* = 3) were incubated for 3 h at 37 °C with the resazurin working solution. At each time point, 100 µL of supernatant from sample or control was loaded into 96-well plates; then, excess supernatant was removed from the cultures and replaced with fresh culture media. The absorbance (λ) of supernatants was measured with a spectrophotometer (Victor 3; PerkinElmer, Waltham, MA, USA) under double-wavelength reading (570 nm and 600 nm). Data were acquired and expressed as the percentage of reduced resazurin by correlating the absorbance values and the molar extinction coefficients of the dye at the selected wavelengths, as shown in the formula:

$$\%Resazurin_{RED}=100\;\times \;\frac{\left(\;117,216\cdot \lambda_{sample@570nm}-\;80,586\cdot \lambda_{sample@600nm}\right)}{\left(155,677\cdot \lambda_{control@600nm}-\;14,652\cdot \lambda_{control@570nm}\right)}$$

At the endpoint, the CM was removed and 1 mL of Calcein AM solution (1 µL/mL of in sterile PBS) was added to the wells and incubated in the dark for 20 min. Calcein AM is a cell-permeant fluorescent dye, which stains the cytoplasm of viable cells in green. Micrographs were acquired under fluorescence mode using a FITC fluorescein filter (Nikon Eclipse Ti, Nikon, Tokyo, Japan).

#### 3.7.6. Indirect Cytotoxicity Tests on Primers

Films of synthesized copolymers P(VAc-co-AA) and P(VA-co-AA) were used to assess cytocompatibility with hDFs. Briefly, the material was soaked in complete CM for 24 h and sterile filtered. HDFs were seeded in 24-well plates at 2 × 10^4^ cells/well and cultured with the conditioned CM and with regular CM as control. Microscopy observations and resazurin assay were performed as described above.

#### 3.7.7. Statistical Analysis

Statistical analyses were carried out by SPSS (SPSS v.16.0; IBM) to specify the significant level of different processing parameters. All the data were analyzed using one-way analysis of variance and Duncan post hoc test for multiple comparisons. Probability (*p*) values <0.05 were considered as statistically significant.

### 3.8. Functional Analysis

Release tests were performed using high-performance liquid chromatography (HPLC, Perkin Elmer Series 200). The systems were placed in tubes containing PBS at 37 °C, under constant stirring. For final coating the release test was carried out also in PBS, with the addition of MMP-9 solution (0.05 μg/mL). At predefined time intervals, the supernatant was collected and analyzed to evaluate the amount of Dexa released. For HPLC analysis the supernatants were placed into 1.5 mL HPLC vials. A 50 µL aliquot of each sample was then injected into the HPLC system. For HPLC analysis, a C18 Phenomenex column, acetonitrile/MilliQ water 70/30 as the mobile phase, UV detector set at 254 nm wavelength, and 1 mL/min eluent flow rate were used. 

### 3.9. Adhesion Evaluation

The adhesion of the composite system to metallic device substrates is a fundamental requirement for the success of the implant. The adhesion test was carried out by immersing the samples (composite coatings on Ti platelets, in triplicate), in wells containing two different solutions: PBS at pH 7.4 and PBS with addition of MMP-9 solution (0.05 μg/mL). The addition of MMP-9 was set in order to simulate a pathological condition, considering that MMP-9 is one of the most frequent proteases associated with the inflammation process in fibrous tissue interfaces [17]. Samples were maintained in a thermostatic bath at 37 °C under continuous stirring. The samples were checked daily over the whole period of analysis (70 days). 

## 4. Conclusions

The realization of a suitable polymeric coating for an implantable device still represents one of main challenges for the success of a permanent implant. Two copolymers, P(Vac-co-AA) and P(VA-co-AA), were synthetized and characterized with the aim to act as a primer, a thin layer at the interface between the metallic surface of device and external coating, to improve the adhesion to device surface. The external layer of PVA hydrogel was produced as a coating of metallic substrate for soft tissue implant. PVA hydrogel was shown to be biocompatible, biostable, and mechanically compatible with soft tissues and able to incorporate and release Dexa. The third component is based on PHBHV; the biopolymer was processed to obtain electrospun fibers showing uniform dimension, smooth surfaces, and capability to incorporate the drug in a homogeneous way. The composite coating showed a good adhesion to titanium substrate, no in vitro cytotoxicity, and a prolonged and controlled drug release. The multifunctional composite coating offers the potential for a long-term interface, able to modulate the release of an anti-inflammatory drugs, thus contrasting acute and chronic inflammation responses following device implantation. 

## Figures and Tables

**Table 1:** Elastic modulus (E’) and loss modulus (E’’) for PVA hydrogel (8% wt).

Sample	E’ (kPa)	E’’ (kPa)
PVA hydrogel	14.4 ± 0.3	0.63 ± 0.32

**Table 2:** Adhesion test for multifunctional coatings on Ti substrate in PBS, with and without MMP-9. The photos were acquired after 70 days of incubation of composite systems in an aqueous media.

Composite Coating with P(VAc-co-AA) Primer in PBS	Composite Coating with P(VAc-co-AA) Primer in PBS + MMP-9	Composite Coating with P(VA-co-AA) Primer in PBS	Composite Coating with P(VA-co-AA) Primer in PBS + MMP-9
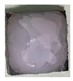	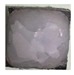	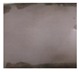	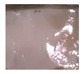
